# Efficiency evaluation of phospholipid fatty acid method based on lipid standards: methanol failed to recover a majority of phospholipids yet eluted unexpected glycolipid

**DOI:** 10.3389/fmicb.2025.1587425

**Published:** 2025-05-14

**Authors:** Shanshan Zhang, Xinyu Jiao, Hongzhang Kang, Wenjuan Yu

**Affiliations:** ^1^State Key Laboratory of Subtropical Silviculture, Zhejiang Agriculture and Forestry University, Hangzhou, China; ^2^College of Chemistry and Materials Engineering, Zhejiang Agriculture and Forestry University, Hangzhou, China; ^3^Department of Landscape Architecture, School of Design, Shanghai Jiao Tong University, Shanghai, China; ^4^Tianmushan Forest Ecosystem National Orientation Observation and Research Station of Zhejiang Province, Hangzhou, China

**Keywords:** phospholipid fatty acid, phospholipids, glycolipids, neutral lipids, lipid standards, acidic and alkaline soils

## Abstract

Phospholipid fatty acid (PLFA) method has been popular and powerful for characterizing soil microbial communities over decades. However, little is known about efficiencies of acidic vs. alkaline extractants and catalysts in PLFA extraction and methylation. More urgently, it remains unclear whether methanol could efficiently elute phospholipids while avoiding recovering non-target lipids. Here, by adding pure lipid standards representative of major neutral, glyco-, and phospholipids into acidic and alkaline soils collected from subtropical China, we comprehensively evaluated efficiencies of extraction, elution, and methylation of three steps in the PLFA method. A good proportion of PLFAs could be extracted by phosphate buffer with both acidic (42–51%) and alkaline (43–68%) soils while citrate buffer worked better for acidic (43–46%) than alkaline (36–47%) soils. Phospholipids were expected to be mainly eluted in methanol, yet we found a non-negligible proportion of phospholipids eluted by chloroform for both acidic (36–71%) and alkaline (9–55%) soils, which is much larger than previously reported. Only 42–50% (acidic soils) and 45–68% (alkaline soils) of phospholipids were recovered in methanol. Meanwhile, 16% (acidic soils) and 5% (alkaline soils) of glycolipid DGDG were unexpectedly eluted into methanol. The alkaline catalyst (mean 86% across all investigated phospholipids) was more efficient in facilitating phospholipids methylation than the acidic one (mean 67%). Overall, incomplete separation among lipid types caused loss of phospholipids and introduction of glycolipid interferences in the methanol fraction, leading to biased estimation of soil microbial biomass and composition. Chloroform elution of phospholipids challenges the principle of “like dissolves like” in solid-phase chromatography, although we cannot rule out the uncertainty caused by background PLFAs and future experiments are needed to provide more evidence. To effectively remove lipid interferences and efficiently elute phospholipids, possible solutions include replacing chloroform with hexane, increasing elution volumes of acetone and methanol, and/or using anion exchange columns.

## 1 Introduction

**Figure 1:**
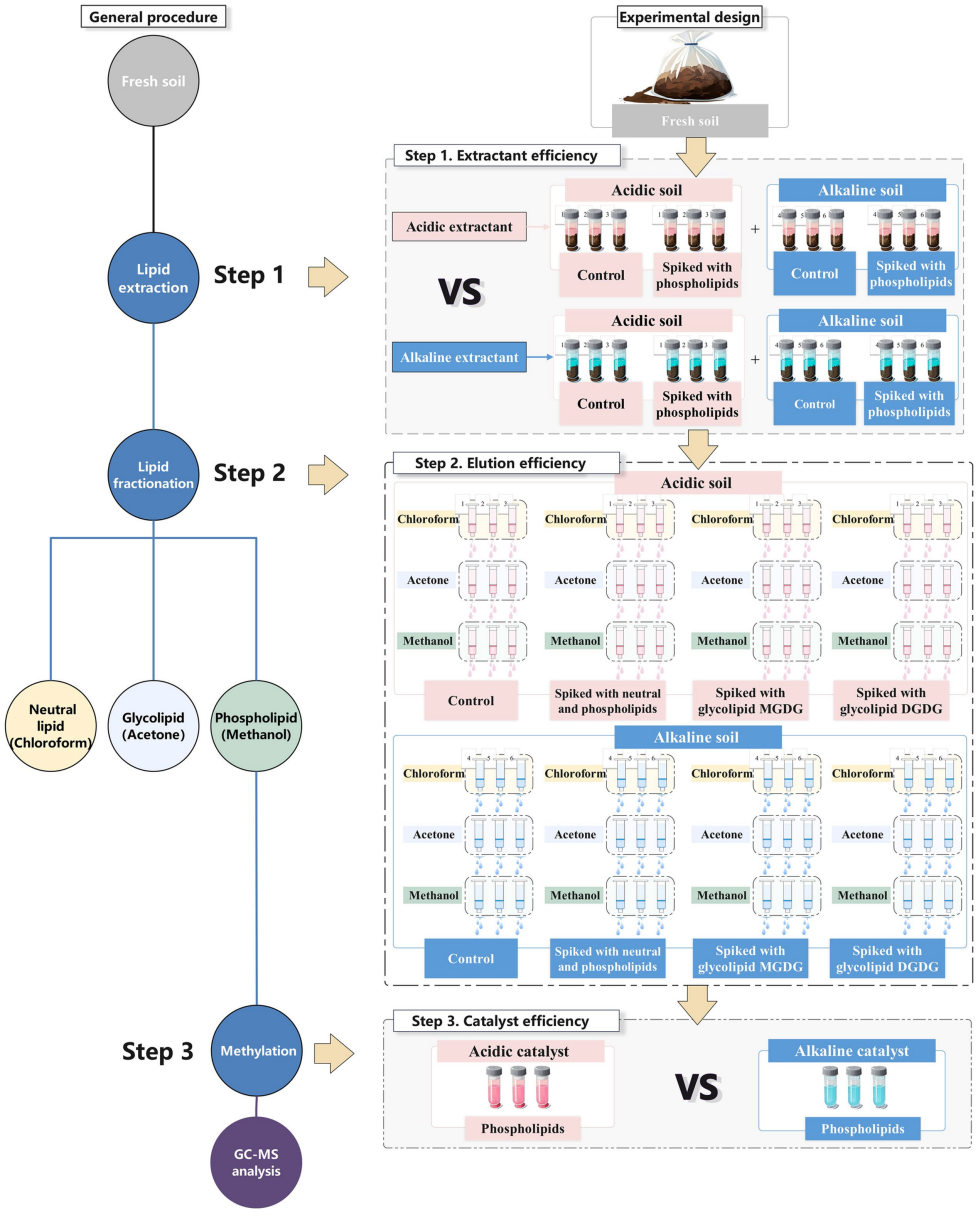
Experimental design of this study. GC–MS, gas chromatography–mass spectrometry.

Phospholipid fatty acid (PLFA) method has been widely used for measure soil microbial biomass and composition (White et al., 1979; Frostegård et al., 1991). This method has been popularly used for more than 40 years as a means to characterize microbial communities in a diverse range of environmental matrices (Willers et al., 2015). Since microbial PLFAs from cell membranes are rapidly degraded upon cell death, they can be considered representative of the viable microbes (White et al., 1979). The method consists of three consecutive steps before gas chromatography analysis (Figure 1). First, microbial lipids are extracted from environmental samples in a mixture of chloroform, methanol, and aqueous buffer. Second, lipids associated with the organic phase are sequentially eluted from silica gel cartridges by solvents of increasing polarity (chloroform, acetone, and methanol), fractionating into neutral, glyco-, and phospholipids, respectively. Finally, the phospholipids in the methanol fraction are subjected to mild methylation to produce fatty acid methyl esters (FAMEs) for gas chromatography analysis. Despite its wide use, the efficiencies of extraction, elution, and methylation from the three steps in the PLFA method has rarely been evaluated in a robust way.

In the first step of lipid extraction, both acidic and alkaline extractants have been used for various soils (Hamer et al., 2007; Fuhrmann et al., 2009). The isolation of lipid material has been achieved by a mixture containing chloroform, methanol and aqueous buffer, which improves contact between extractant and cells in complex matrices and facilitates dissolution of polar lipids in cell membranes (Bligh and Dyer, 1959). Introducing the method into environmental research, White et al. (1979) used a single-phase mixture of chloroform, methanol and phosphate buffer (pH 7.4) to extract phospholipids for determination of sedimentary microbial biomass. Frostegård et al. (1991) reported that an acidic citrate buffer (pH 4.0) gave higher lipid yields from an acid soil with a high organic matter content than the phosphate buffer. Inconsistent findings have been reported among studies comparing efficiencies of acidic vs. alkaline buffers in PLFA extraction for acidic and alkaline soils, yet none of these studies have used lipid standards to unambiguously confirm the efficiency (Nielsen and Petersen, 2000; Papadopoulou et al., 2011).

In the second step of lipid fractionation, chloroform, acetone, and methanol have been used to elute neutral, glyco-, and phospholipids from silica gel solid phase extraction (SPE) cartridges, respectively. Solvent properties, especially its polarity and interaction with lipid groups, significantly influences partitioning of lipids across fractions (Drijber and Jeske, 2019). However, these solvents have increasing rather than contrasting polarity and their elution efficiencies (whether they can efficiently elute the target lipids and avoid eluting the non-target lipids) are questionable. In the very few studies estimating elution efficiency of the popular PLFA methods, less than 10% of phospholipids were eluted by chloroform by estimating P concentration in each fraction or adding standards of various lipid types (Frostegård et al., 1991; Gorka et al., 2023). Yet, a proportion of phospholipids might be eluted by chloroform before methanol, given that chloroform is often used to extract lipids from microorganisms, biological tissues and environmental matrices (Bligh and Dyer, 1959; White et al., 1979; Frostegård et al., 1991) and that chloroform-methanol mixtures could effectively elute phospholipids retained on silica gel cartridges (Suzuki et al., 1993). Besides, using high-performance liquid chromatography–mass spectrometry (LC–MS) to directly detect intact polar lipids, Heinzelmann et al. (2014) showed that the methanol fraction eluted both phospholipids and glycolipids. Thus, the probably incomplete separation among lipid types in current PLFA method may lead to underestimation of PLFAs via their loss in chloroform and overestimation of PLFAs via interference from glycolipids, necessitating a robust examination of elution efficiency of these solvents for neutral, glyco-, and phospholipids.

In the third step of phospholipid methylation, phospholipids are derivatized by methanol to less polar methyl ester derivatives (FAMEs) before gas chromatography analysis. The derivatization reaction is essentially an transesterification process catalyzed by acids or bases (Chowdhury and Dick, 2012). The one-step transesterification has been more commonly used than the multiple-step MIDI method (Schutter and Dick, 2000). The base-catalyzed transesterification method offers advantages of a short reaction time and operation under mild conditions in favor of preserving sensitive fatty acids (Liu et al., 2018). Yet, acidic catalysts can also effectively facilitate transesterification of complex lipids (Christie, 1993), despite the fact that they require heating and longer reaction times than alkaline catalysts. Thus, few studies have compared efficiencies of KOH-vs. HCl-catalyzed phospholipid methylation for acidic soils and concluded that the acid-catalyzed methylation resulted in higher concentrations of most PLFAs than the base-catalyzed one (Chowdhury and Dick, 2012). Further studies based on lipid standards are needed to confirm the conclusion.

Here, by adding pure lipid standards representative of major neutral, glyco-, and phospholipids into acidic and alkaline soils collected from subtropical China, we thoroughly investigated efficiencies of extraction, elution, and methylation of three steps in the PLFA method, with a special focus on examining whether eluents can efficiently elute the target lipids and avoid eluting the non-target lipids in the second step. We aim to (1) compare extraction efficiencies of acidic vs. alkaline extractants in PLFA extraction from soils with contrasting pH values; (2) evaluate efficiencies of chloroform, acetone, and methanol in eluting neutral, glyco-, and phospholipids from silica gel SPE cartridge; (3) compare efficiencies of acidic vs. alkaline catalysts in facilitating PLFA methylation.

## 2 Materials and equipment

In June 2024, we collected three acidic soil samples (pH = 4.68 ± 0.03) from the Tianmushan Forest Ecosystem Research Station in Zhejiang, China (30°20′N, 119°26′E, 338 m above sea level). It has a subtropical humid climate with a mean annual precipitation (MAP) of 1,630 mm and a mean annual temperature (MAT) of 14.9°C (Wu et al., 2025). The soils are Ultisols in the United States Department of Agriculture (USDA) soil classification system. In June 2024, we also collected three alkaline soil samples (pH = 8.24 ± 0.03) from Dongping National Forest Park located on Chongming Island in Shanghai, China (31°41′N, 121°28′E, 3 m above sea level). It is also characterized by a subtropical humid climate, with a mean annual precipitation (MAP) of 1,072 mm and a mean annual temperature (MAT) of 16.6°C (Kang et al., 2018). The soils are Inceptisols. At each site, on each of the three randomly chosen transects, mineral soils at 0–10 cm depth from 10 sampling points (at 5 m spacing) were collected with a 2.5-cm corer and composited, totaling three independent acidic and three independent alkaline soil samples. Soil samples were placed in polyethylene bags, stored on ice and transferred to the laboratory within 24 h. Soils were sieved (2 mm) with any remaining visible plant material and stone removed by hand.

To test efficiency of each step, we added six standards (purity all > 98%) from neutral, glyco-and phospholipids (Table 1; Supplementary Figure S1 for their chemical structure) into the collected soils and evaluated their recovery rates, which we detailed below. These standards cover major soil microbial lipid types. Phosphatidylglycerole (PG), phosphoethanolamine (PE), and phosphatidylcholine (PC) are three major phospholipid classes in soil microbes (Ding et al., 2020); monogalactosyl diglyceride (MGDG) and digalactosyl diglyceride (DGDG) are most abundant glycolipids for soil microbial species (Hölzl and Dörmann, 2007; Ding et al., 2020; Gorka et al., 2023); triacylglycerols and sterols are common neutral lipids for carbon storage in soil microbes (Gorka et al., 2023). Fatty acid composition in these standards could represent, or at least share similar structure with, major soil microbial lipid types because: (1) C16:0 and C18:0 PLFAs are often used for calculating microbial biomass while C18:1ω9 PLFA is used as a fungal biomarker (Zelles, 1997; Bååth, 2003; Kaiser et al., 2010; Kang et al., 2021; Lewe et al., 2021; Yu et al., 2021; Yu et al., 2022); (2) the primary fatty acids found in MGDG and DGDG include C16:0, unsaturated C16 and C18, and possibly C18:0 fatty acids (Jabaji-Hare et al., 1984; Hölzl and Dörmann, 2007; Kostetsky et al., 2018; Ding et al., 2020); (3) while C16:0 and C16:1ω5 fatty acids are dominant for triacylglycerols in arbuscular mycorrhizal fungi, C20:0 fatty acid is also included in soil triacylglycerols (Olsson, 1999; Oates et al., 2017).

**Table 1:** Lipid standards and their chemical information.

Name	Short name	CAS No.	Molecular formula	Fatty acid composition	Lipid group	Provider
Dipalmitoyl Phosphatidylglycerole	DPPG	200880-41-7	C_38_H_74_NaO_10_P	C16:0	Phospholipid	AVT Pharmaceutical Tech (Shanghai)
Dioleoyl Phosphoethanolamine	DOPE	4004-5-1	C_41_H_78_NO_8_P	C18:1ω9	Phospholipid	
Distearoyl Phosphatidylcholine	DSPC	816-94-4	C_44_H_88_NO_8_P	C18:0	Phospholipid	
Glyceryl triarachidate	GTA	620-64-4	C_63_H_122_O_6_	C20:0	Neutral lipid	ANPEL Laboratory Tech (Shanghai)
Monogalactosyl diglyceride	MGDG	41670-62-6	C_45_H_86_O_10_	C18:0	Glycolipid	Sigma-Aldrich Trading (Shanghai)
Digalactosyl diglyceride	DGDG	/	C_51_H_96_O_15_	C18:0	Glycolipid	

DPPG was dissolved in a methanol-chloroform mixture (0.6 mg/mL, 1:2 v/v); DOPE, DSPC, and GTA were dissolved in chloroform (each 0.6 mg/mL); MGDG and DGDG were dissolved in a methanol-chloroform mixture (0.5 mg/mL, 1:4 v/v).

## 3 Methods

### 3.1 General procedure of PLFA method

Lipids were extracted from 10 g of fresh soil using a single-phase mixture of chloroform, methanol, and buffer (Figure 1). Neutral, glyco-and phospholipids were eluted from silica gel SPE cartridge with chloroform, acetone, and methanol, respectively. Phospholipid fatty acids eluted in methanol were converted to FAMEs by a mild methylation and extracted by hexane. The FAMEs were dried at room temperature under nitrogen stream and redissolved in 200 μL of hexane before their analysis on a 7890B Gas Chromatograph/5977B Mass Spectrometer (GC–MS; Agilent, United States). FAMEs were identified by comparing their retention times and mass spectra with those from reference compounds and from the NIST 2014 mass spectral library in the MSD ChemStation software. Methyl nonadecanoate (C19:0 FAME) was used as an internal standard to calculate FAME concentrations.

### 3.2 Comparison of acidic vs. alkaline extractants in step one

To compare efficiencies of acidic vs. alkaline extractants in PLFA extraction from soils with contrasting pH values, we prepared four parallel samples for acidic (two) and alkaline (two) extractants for each of the six fresh soils, totaling 24 samples (Figure 1, first step). For both the two extractants, one was used as a control soil while one was spiked with mixed phospholipid standards (DPPG, DOPE, and DSPC each adding 0.12 mg). A single-phase mixture of chloroform, methanol, and citrate buffer (pH 4.73) and a single-phase mixture of chloroform, methanol, and phosphate buffer (pH 9.37) both in a ratio of 1:2:0.8 were used as acidic and alkaline extractants, respectively. A 16 mL of either acidic or alkaline extractant was added to a soil sample and shook for 2 h at 180 rpm. The supernatant following centrifugation (8,000 rpm, 10 min) was transferred to another glassware tube. The remaining soil was re-extracted using 8 mL of extractant for 1 h and the second supernatant following centrifugation was combined with the first one. Five milliliter of buffer and 5 mL of chloroform were added to the combined supernatant to facilitate separation of chloroform from the other solvents. Lower chloroform phase including extracted lipids was then submitted to lipid fractionation and phospholipid methylation. The recovery rate of lipid extraction could trace how much of the added phospholipid was finally retained following the three steps (Equation 1).


(1) $${\text{Recovery}}_{\text{lipid extraction}}\;(\%)=\frac{\begin{array}{l} \text{mass of lipid in spiked soil}-\\ \text{mass of lipid in control soil} \end{array}}{\text{added lipid mass}}\times 100$$

### 3.3 Evaluation of elution efficiency in step two

To evaluate efficiency of chloroform, acetone, and methanol in eluting neutral, glyco-, and phospholipids from silica gel SPE cartridge, we prepared four parallel soil samples for each of the six fresh soils, totaling 24 soil samples (Figure 1, second step). The first parallel was used as a control soil, the second one was spiked with neutral and phospholipids (GTA, DPPG, DOPE, and DSPC, each adding 0.12 mg), the third one was spiked with 0.10 mg of glycolipid MGDG, and the fourth one was spiked with 0.10 mg of glycolipid DGDG. Following the first extraction step using the alkaline extractant (which we proved was better than the acidic one in the Results and Discussion), the extracted lipid material was redissolved in 2 mL of chloroform and applied on CNW silica gel SPE cartridges (500 mg, 6 mL, 100–200 mesh size, 40–63 μm particle, 60 Å pore diameter, ANPEL Laboratory Tech, Shanghai). The SPE cartridge is widely used for lipid fractionation in PLFA analysis (Frostegård et al., 1991; Quideau et al., 2016) and a capacity of 500 mg silica gel is sufficient enough to adsorb original and added microbial lipids. The cartridges were eluted with 4 mL of chloroform, 8 mL of acetone, and 8 mL of methanol, targeting neutral, glyco-, and phospholipids, respectively. The lipids eluted in different solvents were dried at room temperature under nitrogen stream and redissolved in 1 mL of methanol:toluene (1:1 v) for base-catalyzed methylation (which would rule out interferences from free fatty acids), totaling 72 samples for GC–MS analysis. The recovery rate of a lipid in an eluent could trace how much of the added lipid was eluted by the solvent in the second step (Equation 2).


(2) $${\text{Recovery}}_{\text{lipid in eluent}}\;(\%)=\frac{\begin{array}{l} \text{mass of lipid collected in}\;\\ \text{eluent in spiked soil}-\\ \text{mass of lipid collected in}\;\\ \text{eluent in control soil} \end{array}}{\text{added lipid mass}}\times 100$$

### 3.4 Comparison of acidic vs. alkaline catalysts in step three

To compare efficiencies of acidic vs. alkaline catalysts in facilitating PLFA methylation, we prepared six replicates of mixed phospholipid standards (DPPG, DOPE, and DSPC, each adding 0.04 mg), three for acidic and three for alkaline catalysts (Figure 1, step 3). A sulfuric acid solution (5% in methanol) and a potassium hydroxide solution (0.2 mol/L in methanol) were used as acidic and alkaline catalysts, respectively. In the base-catalyzed methylation, phospholipid standards were thoroughly mixed with 1 mL of methanol:toluene (1:1 v/v) and 1 mL of catalyst in a glass tube. After incubating the solution at 37°C for 0.5 h, 0.25 mL of acetic solution (1 mol/L in water) was added to end the methylation reaction. Following addition of 2 mL of hexane and 2 mL of water, the solution was vortexed for 2 min and centrifuged (2,600 rpm, 10 min). The upper hexane phase containing FAMEs were transferred to an empty glass tube. The step was repeated once without water and the two hexane phases were combined. The FAMEs were dried at room temperature under nitrogen stream and redissolved in 200 μL of hexane before their analysis on GC–MS. In the acid-catalyzed methylation, most steps were repeated except that the reaction was conducted at 60°C overnight on a water bath, the acetic solution was not added, and the combined hexane portions were washed with 2% KHCO_3_ to neutralize any remaining acid. The recovery rate of a lipid following an acid-or base-catalyzed methylation could trace how much of the added phospholipid was converted into FAME by the catalyst in the third step (Equation 3).


(3) $${\text{Recovery}}_{\text{lipid methylation}}\;(\%)=\frac{\begin{array}{l} \text{mass of lipid after}\;\\ \text{catalyzed methylation} \end{array}}{\text{added lipid mass}}\times 100$$

### 3.5 Statistical analysis

Mean and standard error (*N* = 3) of recovery rates in each step were calculated and presented in R statistical software version 3.6.1 (R Core Team, 2019). One-way ANOVA analysis was used to test if recovery rates significantly (*p* < 0.05) differed between extractants and catalysts with contrasting pH values.

## 4 Results and discussion

### 4.1 The alkaline extractant had higher efficiencies in extracting phospholipids than the acidic one

**Figure 2:**
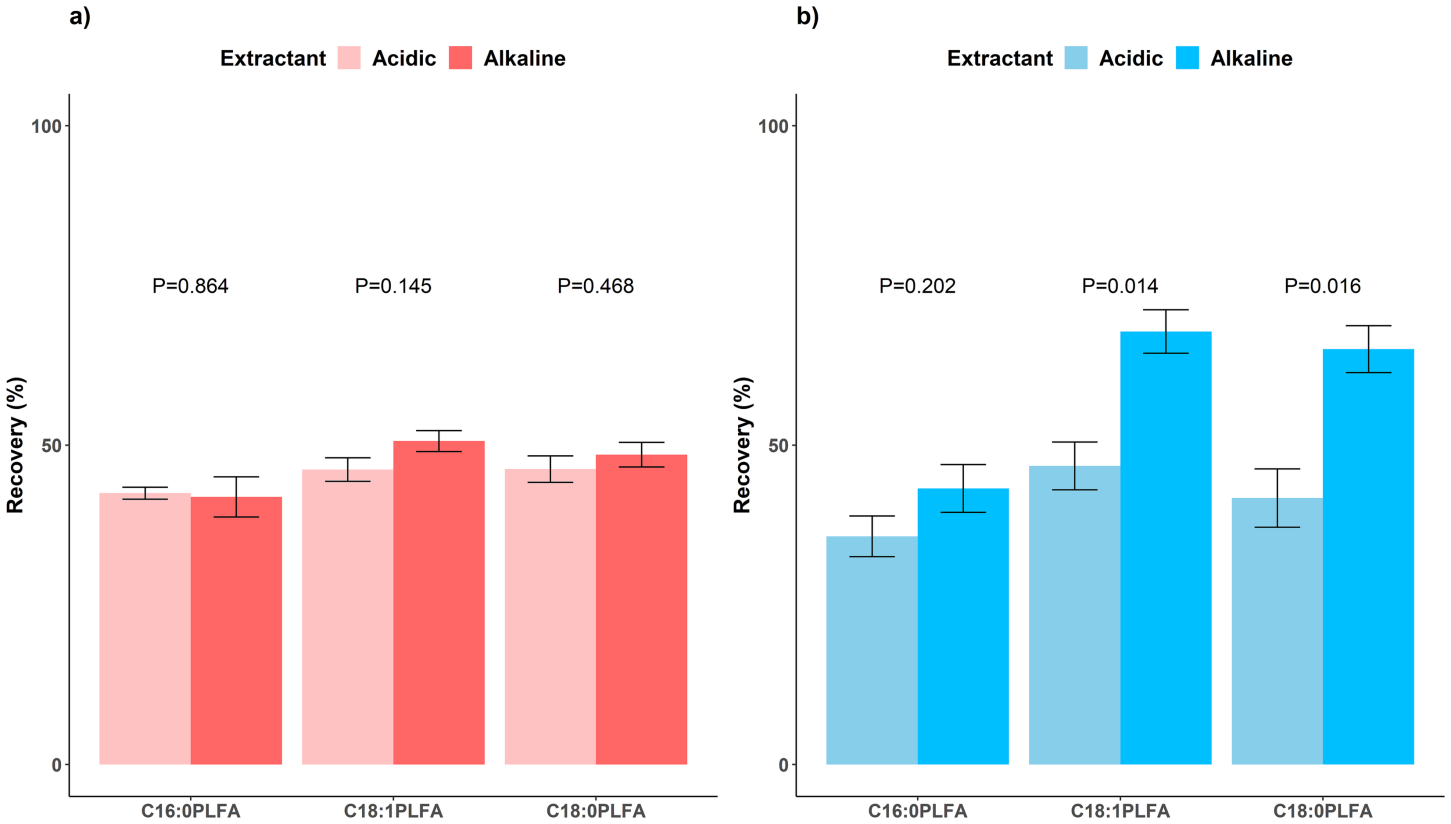
Recovery rates of phospholipids extracted by acidic (light) and alkaline (dark) extractants for **(a)** acidic (red, left) and **(b)** alkaline (blue, right) soils. Means and error bars (*N* = 3) are presented. *p* values showing whether recovery rates between acidic and alkaline extractants were significant (*p* < 0.05) are presented above columns.

For acidic soils (Figure 2a), 42.5 ± 0.9%, 46.2 ± 1.8%, and 46.2 ± 2.1% of C16:0, C18:1, and C18:0 phospholipids were extracted by the acidic extractant, respectively; 41.9 ± 3.1%, 50.6 ± 1.6%, and 48.5 ± 1.9% of C16:0, C18:1, and C18:0 phospholipids were extracted by the alkaline extractant, respectively; no significant (P all > 0.05) differences in recovery were observed between acidic and alkaline extractants. For alkaline soils (Figure 2b), 35.7 ± 3.2%, 46.7 ± 3.7%, and 41.7 ± 4.6% of C16:0, C18:1, and C18:0 phospholipids were extracted by the acidic extractant, respectively; 43.2 ± 3.7%, 67.8 ± 3.4%, and 65.0 ± 3.7% of C16:0, C18:1, and C18:0 phospholipids were extracted by the alkaline extractant, respectively; the alkaline extractant had significantly (*p* < 0.05) higher extraction efficiencies than the acidic one for both C18:1 (*p* = 0.014) and C18:0 (*p* = 0.016) phospholipids.

Following the usage of an alkaline extractant consisting of chloroform, methanol, and phosphate buffer for PLFA extraction from environmental samples recommended by White et al. (1979), Frostegård et al. (1991) proposed an acidic extractant consisting of chloroform, methanol, and citrate butter for PLFA extraction in acidic soils with high organic matter contents. Previous studies have compared PLFA concentrations obtained by acidic vs. alkaline extractants for acidic and alkaline soils (Nielsen and Petersen, 2000; Papadopoulou et al., 2011), making inconsistent conclusions. By adding pure lipid standards, our study confirmed that a good proportion of PLFAs could be extracted by phosphate buffer with both acidic and alkaline soils while citrate buffer worked better for acidic than alkaline soils. Thus, we recommend using phosphate buffer to extract microbial PLFAs from both acidic and alkaline soils while using citrate buffer for acidic soils only. This conclusion has implications for broader scales and other ecosystems including agriculture, grassland, and wetland, although more experiments are needed.

### 4.2 Methanol did not recover a majority of phospholipids yet eluted unexpected glycolipid

**Figure 3:**
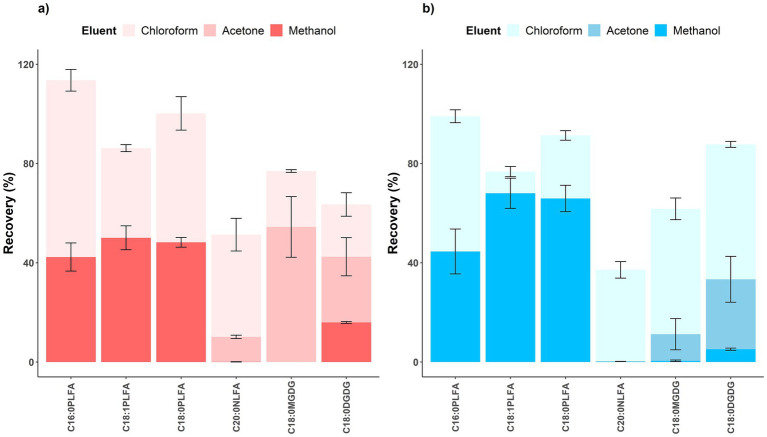
Recovery rates of lipids sequentially eluted in chloroform (light), acetone (medium), and methanol (dark) in step two for **(a)** acidic (red, left) and **(b)** alkaline (blue, right) soils. Means and error bars (*N* = 3) are presented. NLFA, neutral lipid fatty acids. Notedly, negative recovery values were reported for acetone elution of phospholipids in both acidic and alkaline soils, but these negative values were not reflected to avoid confusion.

Phospholipids were expected to be eluted in methanol, yet we found a non-negligible proportion of phospholipids eluted by chloroform for both acidic (36.1–71.2%) and alkaline (8.7–54.5%) soils. For acidic soils, 42.3 ± 5.7%, 50.1 ± 4.8%, and 48.3 ± 2.0% of C16:0, C18:1, and C18:0 phospholipids were recovered in methanol, respectively; 71.2 ± 4.4%, 36.1 ± 1.4%, and 51.9 ± 6.7% of C16:0, C18:1, and C18:0 phospholipids were eluted in chloroform, respectively (Figure 3a). Similarly for alkaline soils, 44.6 ± 9.0%, 68.0 ± 6.1%, and 65.9 ± 5.3% of C16:0, C18:1, and C18:0 phospholipids were recovered in methanol, respectively; 54.5 ± 2.6%, 8.7 ± 2.1%, and 25.4 ± 1.9% of C16:0, C18:1, and C18:0 phospholipids were eluted in chloroform, respectively (Figure 3b). None of the phospholipids were eluted in acetone as expected. Notedly, negative recovery values were reported for acetone elution of phospholipids in both acidic and alkaline soils, probably due to experimental errors and similarity between spiked and control soils in the nonnegligible concentrations of other lipids recovered in acetone (Supplementary dataset), but these negative values were not reflected in Figure 3 to avoid confusion. Consistent with elution of phospholipids from silica gel cartridges by chloroform-methanol mixtures (Suzuki et al., 1993), our finding reports a much larger proportion (8.7–71.2%) of phospholipids eluted in chloroform than that (<10%) in few studies showing the recovery data (Frostegård et al., 1991; Gorka et al., 2023).

Moreover, similar to the findings with a marine sediment (Heinzelmann et al., 2014), we found that the recovery in methanol varied among phospholipids (42.3–50.1% for acidic soils and 44.6–68.0% for alkaline soils), probably due to their polarity difference. Over half of C16:0 PLFA was eluted by chloroform for both acidic and alkaline soils, because it is less polar with a hydrophilic end of glycerol and more affinitive to chloroform. In contrast, relatively larger proportions of C18:1 and C18:0 PLFAs were eluted by methanol, especially for alkaline soils, as C18:1 PLFA with an ethanolamine end and C18:0 PLFA with a choline end are more polar (Figure 3; Supplementary Figure S1; Dufourcq and Lussan, 1972). The experimental results could thus reflect PLFA concentrations to different extents, leading to less accurate characterization of soil microbial biomass and composition and interpretation of their relationships with biogeochemical variables (Heinzelmann et al., 2014).

On the other hand, glycolipid C18:0 DGDG was partly eluted by methanol, bringing interference to quantification of phospholipid markers, especially in acidic soils. For glycolipid C18:0 MGDG, 54.4 ± 12.2% were eluted in acetone and 22.5 ± 0.5% in chloroform for acidic soils and 10.7 ± 6.3% were eluted in acetone and 50.5 ± 4.4% in chloroform for alkaline soils; its elution by methanol was negligible (Figure 3). Yet for glycolipid C18:0 DGDG, while 26.5 ± 7.7% (acidic soil) and 28.2 ± 9.2% (alkaline soil) were eluted by acetone and 21.1 ± 4.7% (acidic soil) and 54.4 ± 1.2% (alkaline soil) were eluted by chloroform, a proportion was also eluted by methanol (15.9 ± 0.4% for acidic soils and 5.2 ± 0.4% for alkaline soils). Consistently, Heinzelmann et al. (2014) used LC–MS for direct detection of intact polar lipids to show that the methanol fraction contained not only phospholipids but also other lipid classes including glycolipids (MGDG and DGDG). The phospholipid and glycolipid fractions contained ca. 50 and 14% of total microbial lipids in forest soils, respectively (Gorka et al., 2023). Combining this study with our data (16% of glycolipid DGDG and ca. 50% of phospholipids eluted in methanol for acidic soils; Figure 3a), in worse-case scenario, glycolipid could interfere with microbial biomass quantification by up to 8%, according to the following Equation 4.


(4) $$\begin{array}{l} \text{Interference of glycopid to phospholipid}=\\ \frac{0.16\times 0.14\;\text{glycolipid}}{0.16\times 0.14\;\text{glycolipid}+0.5\times 0.5\;\text{phospholipid}} \end{array}$$

It is reassuring to find that neutral lipid C20:0 GTA was successfully removed by chloroform (41.2 ± 6.6%) and acetone (10.1 ± 0.7%) for acidic soils and thoroughly removed by chloroform (36.9 ± 3.3%) in alkaline soils (Figure 3). Negligible proportions of the neutral lipid were recovered in methanol for both acidic and alkaline soils. Chloroform thus proved efficient in removing neutral lipids. Throughout the three steps, the un-recovered proportions of the added lipids were possibly lost by adsorption to soil matrix, non-adsorption by silica gel cartridges before elution (especially for neutral and glycolipids), elution by other solvents, and insufficient methylation. Overall, non-negligible proportions of phospholipids were eluted in chloroform before methanol (especially for acidic soils and C16:0 PLFA with a glycerol end) while a considerable part of glycolipid (C18:0 DGDG) was eluted by methanol, both leading to inaccurate estimation of soil microbial biomass and composition due to either loss of phospholipids or introduction of glycolipid interferences.

Future studies are urgently needed to find solvent alternatives or modify SPE columns that could effectively remove major interferences and exclusively elute phospholipids. We propose two potential ways. First, using alternative solvents along with modified volumes to elute lipids from silica gel SPE column. Consider using 4 mL of hexane or petroleum ether to fully elute triacylglycerols, then using 10 mL of acetone or more to fully elute glycolipids, finally using 10 mL of methanol or more to fully elute phospholipids. In this proposed method, we will discard chloroform since it unexpectedly eluted a non-negligible part of both glycolipids and phospholipids. Hexane and petroleum ether (similar to ethyl ether but less harmful) have been proved to effectively elute nonpolar triacylglycerols but not more polar lipids (Danielewicz et al., 2011). Acetone with moderate polarity seems to be appropriate for eluting glycolipids also with moderate polarity, and glycolipids are not expected to be pre-eluted by nonpolar hexane, but the elution volume needs to be increased in order for glycolipids to be fully eluted by acetone before methanol, so as to eliminate the interference of glycolipids to phospholipids. Similarly, polar methanol seems to be appropriate for eluting polar phospholipids which are not expected to be pre-eluted by nonpolar hexane, and the elution volume could be increased in order for phospholipids to be fully eluted. Additionally, using representative lipid standards covering diverse structure and polarity for each of the major lipid types, we will test the proposed solvents and elution volumes using thin layer chromatography and adjust if needed, before formal separation using SPE columns.

Second, to use an anion exchange column for lipid separation. Ion-exchange columns are widely used for purification of proteins and nucleic acids including acidic and alkaline groups, separating them based on their net surface charge, which can be manipulated by pH and ionic strength conditions (Fioretti et al., 2022). Phospholipids also including both acidic and alkaline groups show a net negative charge under mild alkaline pH and should be easily retained on the column, while uncharged glyco-and neutral lipids pass through (Domonkos et al., 2008; Naumowicz et al., 2020). Thus, anion exchange columns along with mild alkaline conditions are expected to separate phospholipids from other lipids, and experiments are needed to confirm this hypothesis.

### 4.3 The alkaline catalyst had higher efficiencies in phospholipid methylation than the acidic one

**Figure 4:**
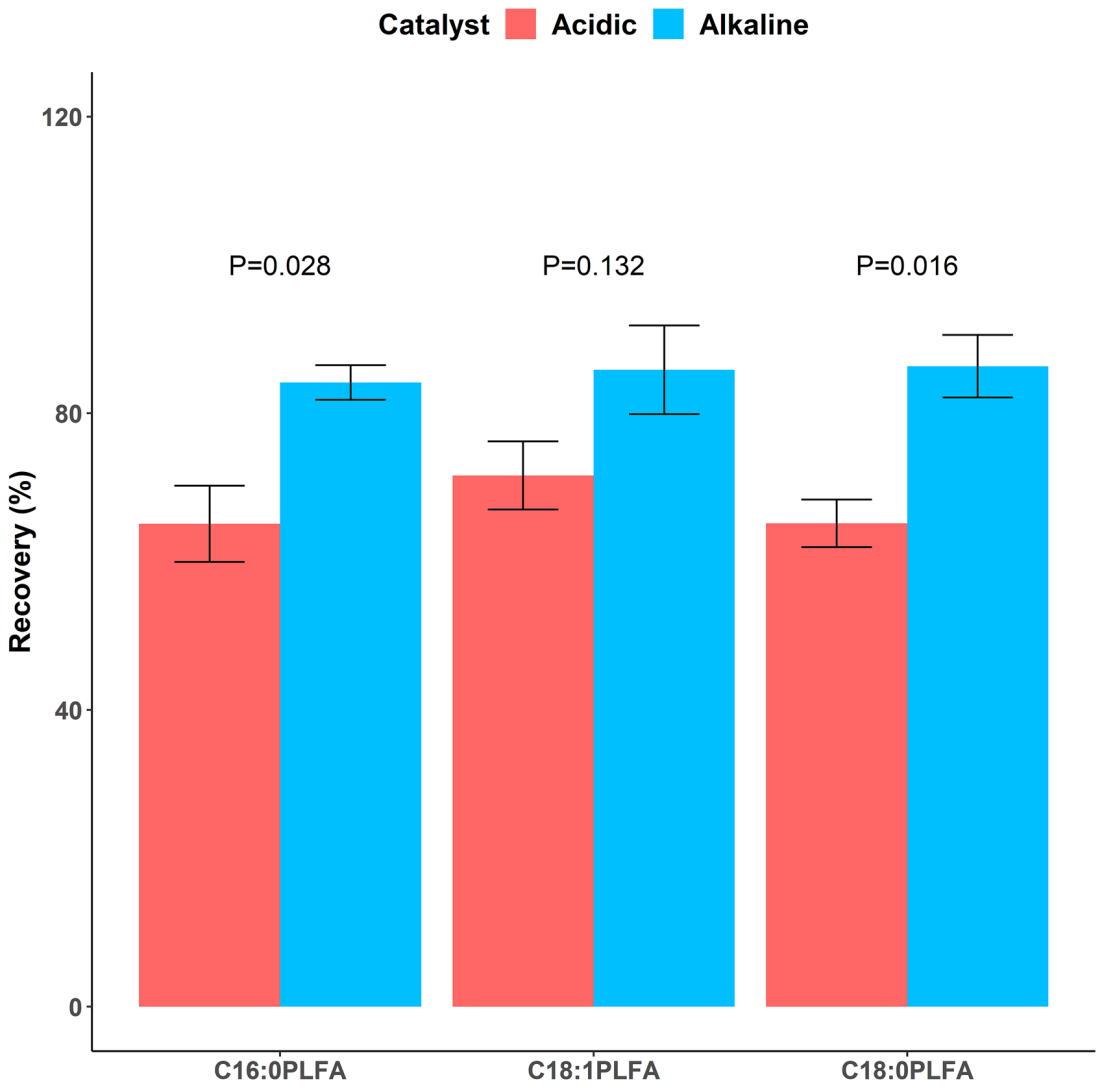
Recovery rates of phospholipids following methylation facilitated by acidic (dark red) and alkaline (dark blue) catalysts. Means and error bars (*N* = 3) are presented. *p* values showing whether recovery rates between acidic and alkaline catalysts were significant (*p* < 0.05) are presented above columns.

65.1 ± 5.1%, 71.6 ± 4.6%, and 65.2 ± 3.2% of C16:0, C18:1, and C18:0 phospholipids were converted into FAMEs with the addition of the acidic catalyst, respectively; 84.2 ± 2.3%, 85.9 ± 6.0%, and 86.3 ± 4.2% of C16:0, C18:1, and C18:0 phospholipids were converted into FAMEs with the addition of the alkaline catalyst, respectively (Figure 4). The alkaline catalyst had significantly (*p* < 0.05) higher catalytic efficiencies than the acidic one for C16:0 (*p* = 0.028) and C18:0 (*p* = 0.016) phospholipids. Inconsistent with a study reporting acid-catalyzed methylation resulted in higher concentrations of most PLFAs than base-catalyzed one (Chowdhury and Dick, 2012), our study used lipid standards to show that the alkaline catalyst (mean 85.5% across all investigated phospholipids) was more efficient in facilitating conversion of phospholipids into FAMEs than the acidic one (mean 67.3%). Further considering its convenience (performed at room temperature), we thus recommend using mild base-catalyzed methylation in the third step of the PLFA method.

## 5 Conclusion

In summary, by adding lipid standards representing major phospholipids, glycolipids and neutral lipids into acidic and alkaline soils, we thoroughly examined extraction, elution, and methylation efficiencies of three steps in the PLFA method, which has been rarely evaluated before. Background lipid interference from control soils was not a big issue as lipid level in the spiked sample was usually higher than that in the control sample (Supplementary dataset). Meanwhile, C16:0 (5.12 μg/g) and C18:1 (4.74 μg/g) PLFAs in control acidic soils were comparable to the spiked concentrations of C16:0 (17.40 μg/g) and C18:1 (19.10 μg/g) PLFAs, reflecting that the spiked concentrations could sometimes reflect realistic conditions. SPE cartridge was not very likely to be overloaded as 500 mg of silica gel is sufficient enough to adsorb lipids both from soils and standards. Recovery of the added lipids confirmed a higher extraction efficiency of phosphate buffer and a higher methylation efficiency of alkaline catalyst than their acidic counterparts. Most importantly, the methanol fraction in the elution step contained fatty acids derived from glycolipids, interfering with PLFA quantification by up to 8% by a rough estimation, while missing a considerable part of phospholipids that had been eluted by chloroform. The proportion of phospholipids eluted in chloroform is reasonable and much larger than previously reported. Chloroform elution of phospholipids challenges the principle of “like dissolves like” in solid-phase chromatography, although we cannot rule out the uncertainty caused by background PLFAs and experiments including LC–MS are needed to provide more unambiguous evidence in the future. The incomplete separation among soil lipids might lead to biased evaluation of soil microbial biomass and composition. We propose that in order to effectively remove lipid interferences and efficiently elute phospholipids, future solutions could consider replacing chloroform with hexane, increasing elution volumes of acetone and methanol, and/or using anion exchange columns.

## Data Availability

The original contributions presented in the study are included in the article/Supplementary material, further inquiries can be directed to the corresponding author.
