# Supplementary material for: Efficiency evaluation of phospholipid fatty acid method based on lipid standards: methanol failed to recover a majority of phospholipids yet eluted unexpected glycolipid
Source: Front Microbiol. 2025 May 14;16:1587425. doi: 10.3389/fmicb.2025.1587425 (PMC12116389; doi:10.3389/fmicb.2025.1587425)
Supplement: Supplementary file 1 [file Supplementary_file_1.zip › Supplementary Figure S1.DOCX]

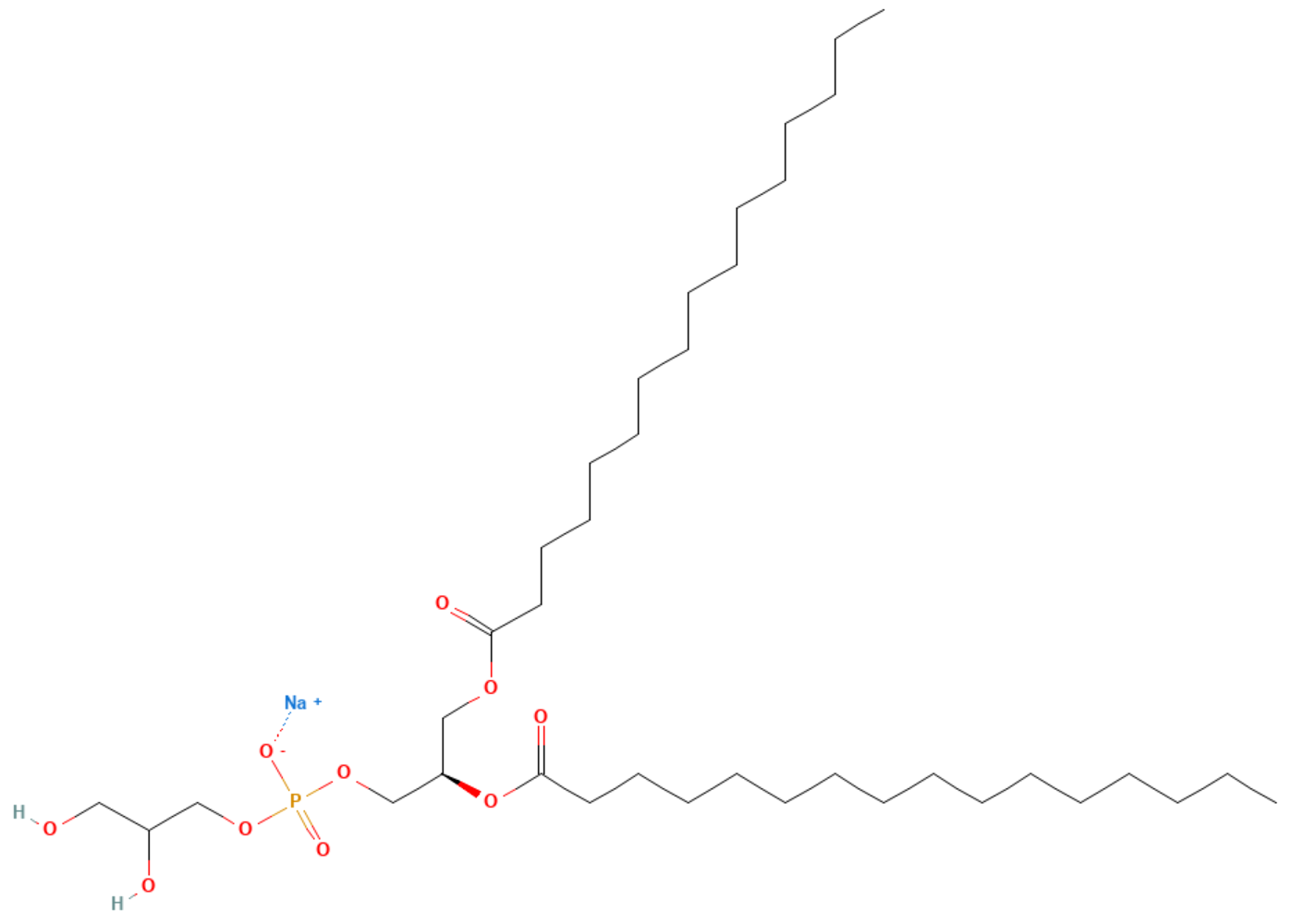


DPPG, C16:0 phospholipid


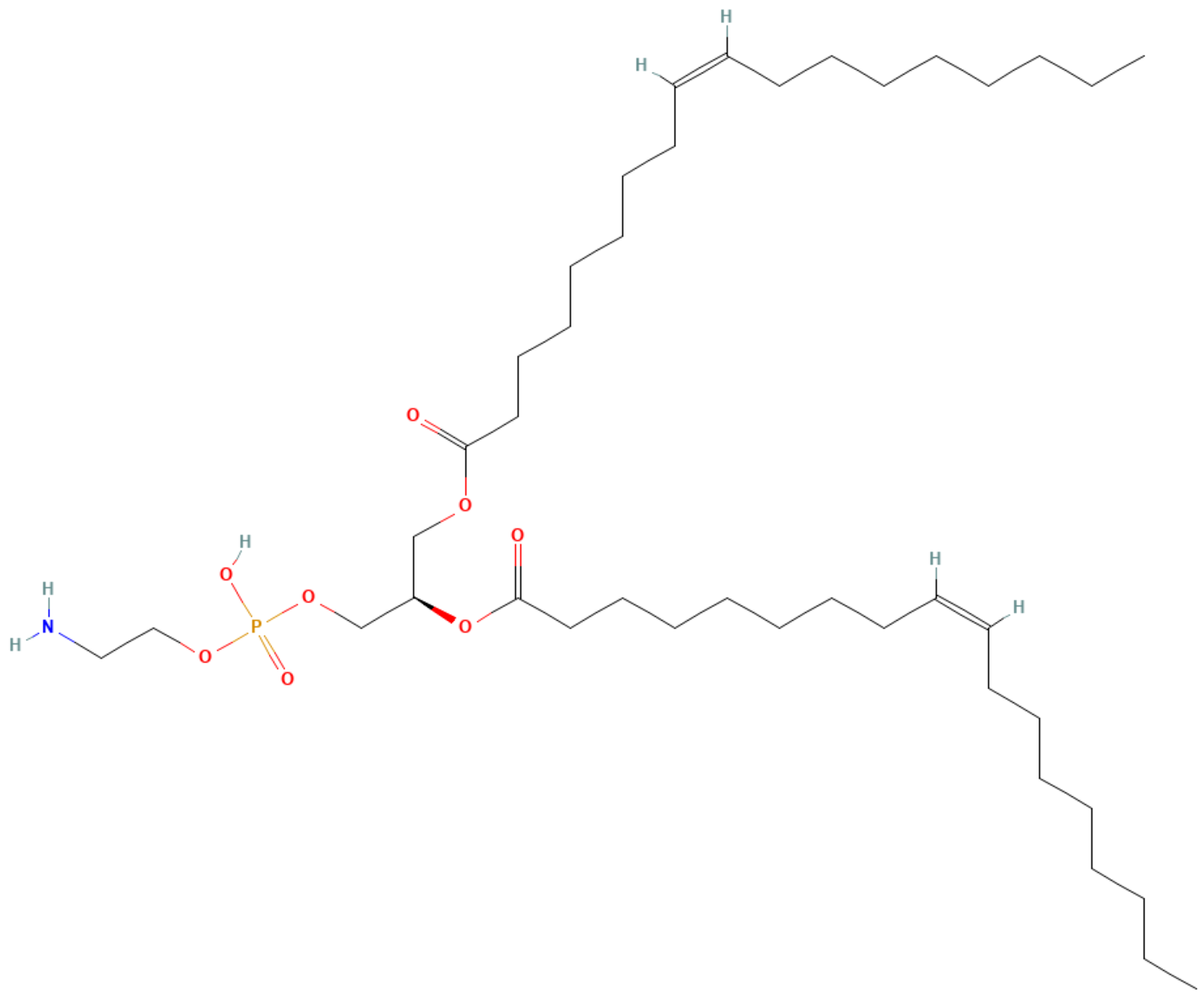


DOPE, C18:1 phospholipid


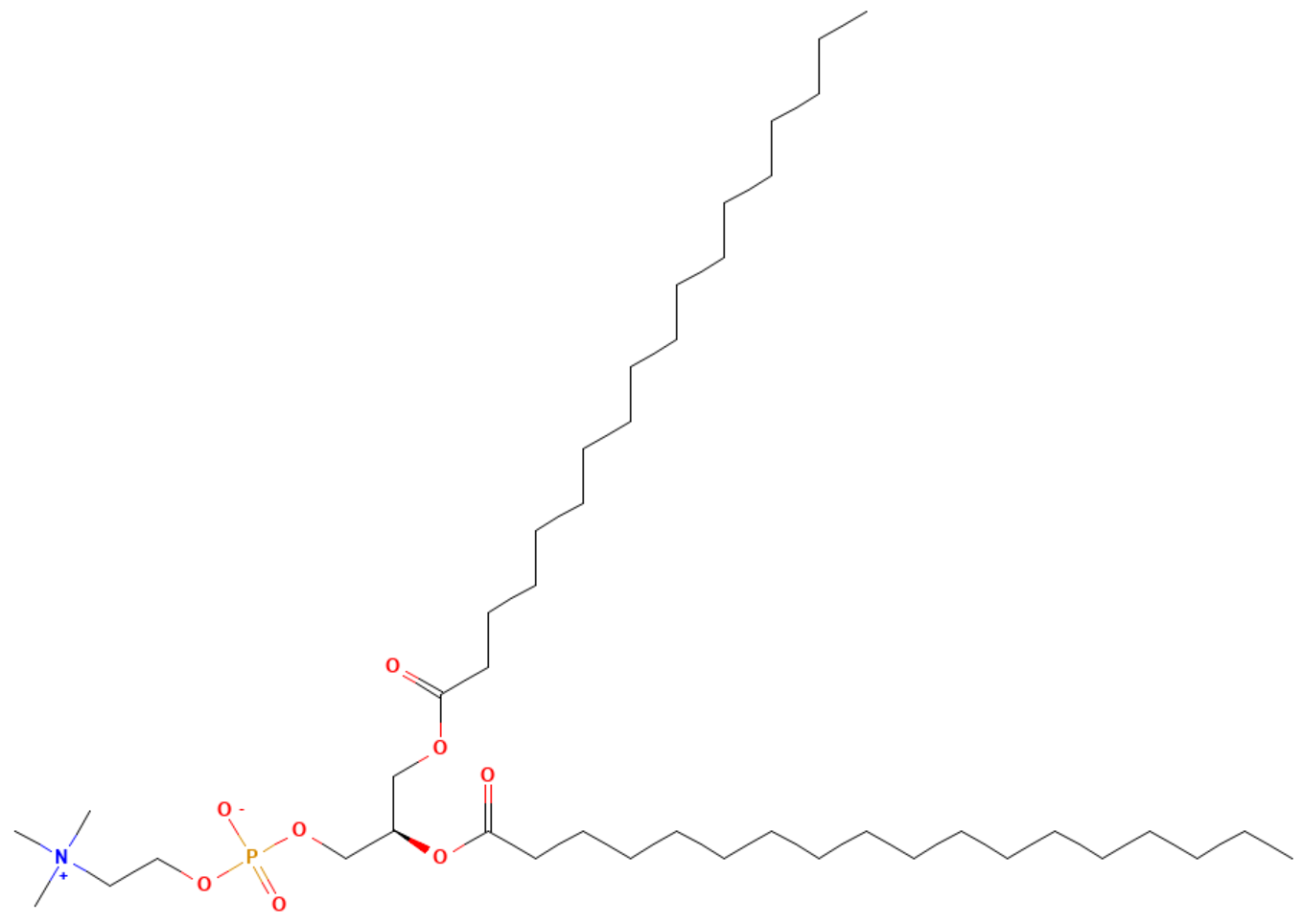


DSPC, C18:0 phospholipid


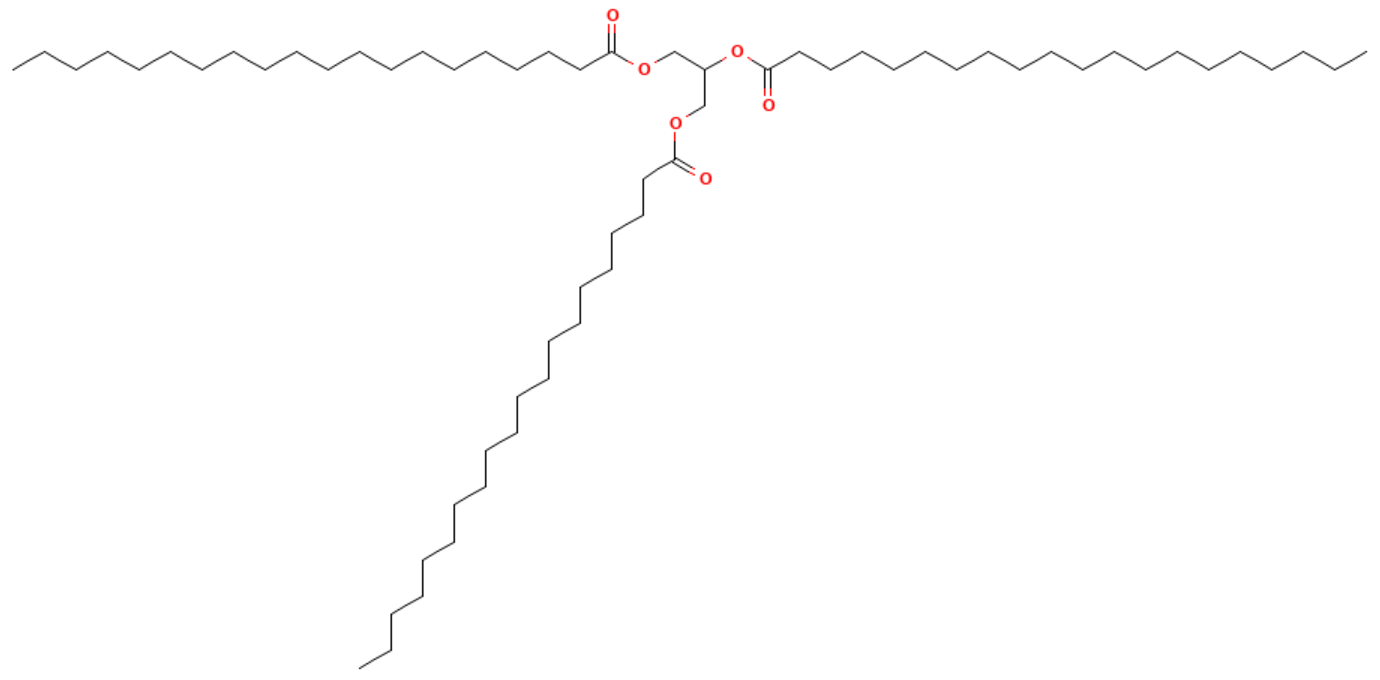


GTA, C20:0 neutral lipid


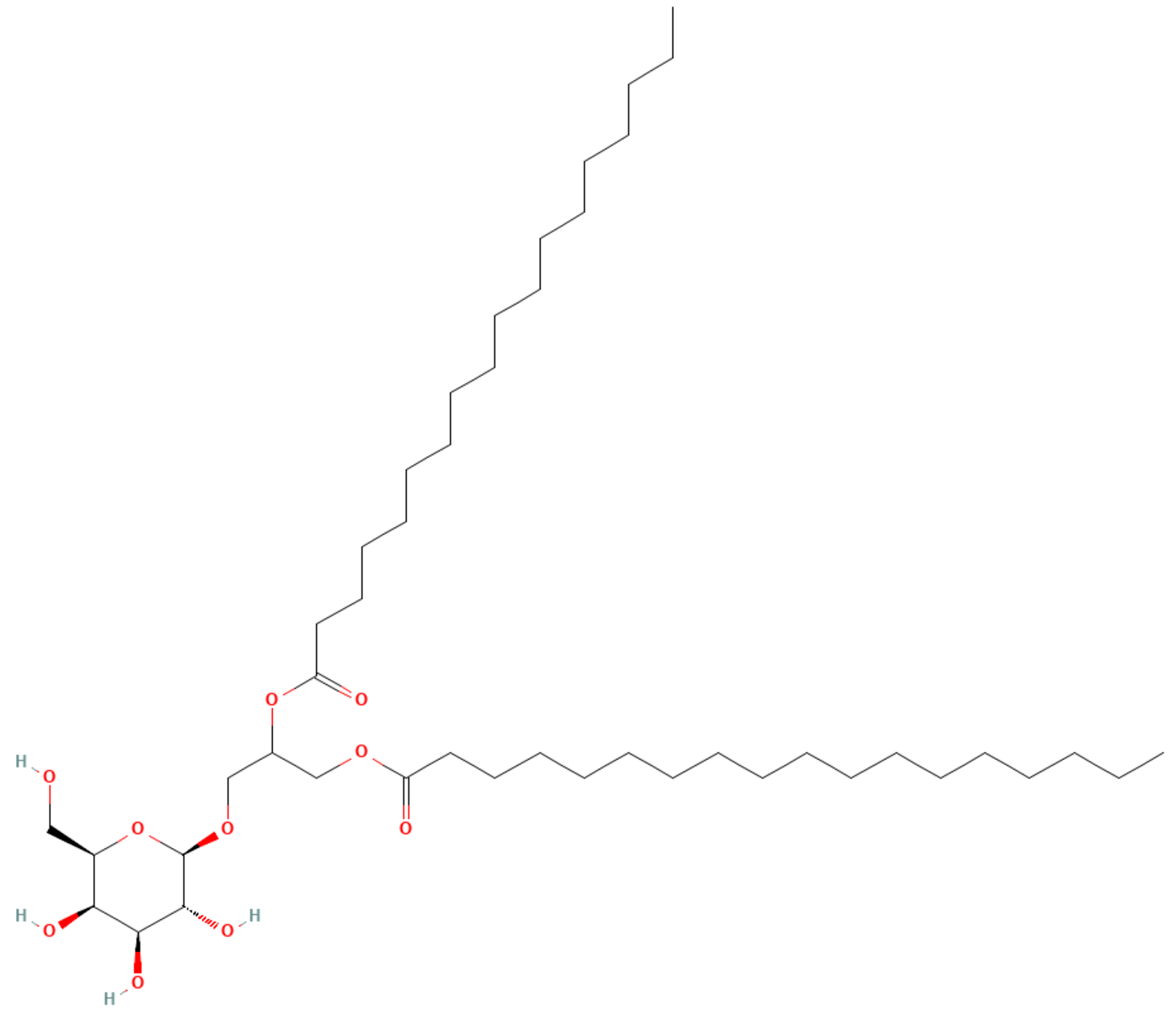


MGDG, C18:0 glycolipid


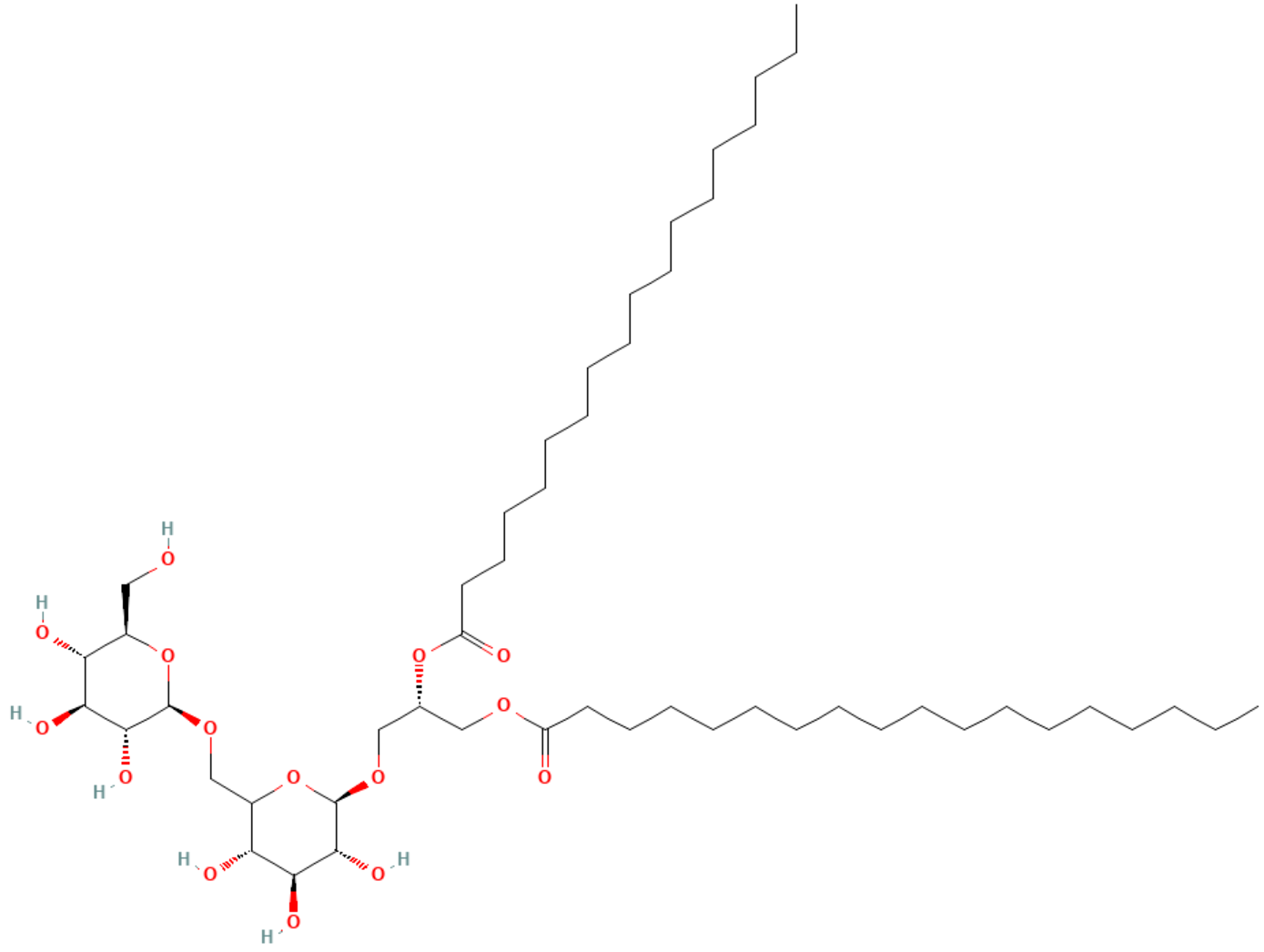


DGDG, C18:0 glycolipid

**Fig. S1** Chemical structures of the six lipid standards
